# Disentangling the drivers of taxonomic and phylogenetic beta diversities in disturbed and undisturbed subtropical forests

**DOI:** 10.1038/srep35926

**Published:** 2016-10-24

**Authors:** Jinliang Liu, Hong Qian, Yi Jin, Chuping Wu, Jianhua Chen, Shuquan Yu, Xinliang Wei, Xiaofeng Jin, Jiajia Liu, Mingjian Yu

**Affiliations:** 1 College of Life Sciences, Zhejiang University, Hangzhou, Zhejiang 310058, China; 2 Research and Collections Center, Illinois State Museum, 1011 East Ash Street, Springfield, IL 62703, USA; 3 Zhejiang Forestry Academy, Hangzhou, Zhejiang 310023, China; 4 College of Chemistry and Life Sciences, Zhejiang Normal University, Jinhua, Zhejiang 321004, China; 5 School of Forestry and Bio-technology, Zhejiang A&F University, Lin’an, Zhejiang 311300, China; 6 School of Environmental & Resource Sciences, Zhejiang A&F University, Lin’an, Zhejiang 311300, China; 7 College of Life and Environmental Sciences, Hangzhou Normal University, Hangzhou, Zhejiang 311121, China

## Abstract

Understanding the relative importance of dispersal limitation and environmental filtering processes in structuring the beta diversities of subtropical forests in human disturbed landscapes is still limited. Here we used taxonomic (TBD) and phylogenetic (PBD), including terminal PBD (PBDt) and basal PBD (PBDb), beta diversity indices to quantify the taxonomic and phylogenetic turnovers at different depths of evolutionary history in disturbed and undisturbed subtropical forests. Multiple linear regression model and distance-based redundancy analysis were used to disentangle the relative importance of environmental and spatial variables. Environmental variables were significantly correlated with TBD and PBDt metrics. Temperature and precipitation were major environmental drivers of beta diversity patterns, which explained 7–27% of the variance in TBD and PBDt, whereas the spatial variables independently explained less than 1% of the variation for all forests. The relative importance of environmental and spatial variables differed between disturbed and undisturbed forests (e.g., when Bray-Curtis was used as a beta diversity metric, environmental variable had a significant effect on beta diversity for disturbed forests but had no effect on undisturbed forests). We conclude that environmental filtering plays a more important role than geographical limitation and disturbance history in driving taxonomic and terminal phylogenetic beta diversity.

Species composition in assemblages is driven by both ecological and evolutionary factors^1^,^2^. Accordingly, beta diversity (i.e., species turnover) between assemblages is also driven by ecological and evolutionary factors. Taxonomic beta diversity (TBD) measures differences in species composition between assemblages without taking into account evolutionary histories of species because TBD treats all species equally. In contrast, phylogenetic beta diversity (PBD) quantifies differences in species composition between assemblages in an evolutionary framework (i.e., phylogeny). TBD and PBD between assemblages are determined by environmental difference, which is relevant to environmental filtering, and geographical distance, which is relevant to dispersal limitation. Unraveling the relative importance of environment and space on TBD and PBD patterns is critical to understanding the roles played by historical and current ecological processes in shaping the regional biodiversity^3^,^4^,^5^,^6^. The impacts of these drivers on TBD along spatial and environmental gradients have been well documented^3^,^7^,^8^,^9^, but TBD analysis alone cannot detect the effects of evolutionary processes on community assembly^10^. Instead, analysis on PBD, which measures the changes in community phylogenetic compositions between sites, takes into account evolutionary history when addressing the issue of beta diversity^10^,^11^,^12^,^13^,^14^.

Phylogenetic beta diversity may be driven by turnover at shallow nodes such as nodes leading to terminal branches in a phylogeny; alternatively, it may be driven by turnover at deep nodes such as basal nodes of the phylogeny. Terminal and basal phylogenetic beta diversities (PBDt and PBDb, respectively) measure phylogenetic turnover in community composition at different depths of evolutionary history^15^. Specifically, PBDt measures the phylogenetic turnover among recently diverged lineages, while PBDb measures the turnover deep within phylogeny. Selective pressures may favor traits expressed within certain phylogenetic lineages under a strong environmental filtering. For example, if turnover of species within clades is weaker than turnover deep within phylogeny, then entire clades track environmental conditions; otherwise, if turnover of species within clades is greater than turnover at deeper levels, selective pressures promote divergence into habitats, and thus recently evolved species are likely to occupy different environmental regimes than their ancestors^16^. Using both PBDt and PBDb measures might help us to understand whether the phylogenetic divergence between an array of sites has occurred recently or deep in the past.

Environmental filtering and dispersal limitation are major drivers generating patterns of TBD and PBD^3^,^17^. However, the relative importance of dispersal limitation and environmental filtering varies with study system and spatial scale^6^,^18^,^19^,^20^. Previous studies showed that dispersal limitation dominates at local and intermediate scales^4^,^21^ while environmental filtering dominates at large scales^11^,^19^. Further, different patterns of beta diversity in different regions may reflect different mechanisms of community assembly; for example, environmental filtering was often found to be more important in temperate forests than in tropical forests^3^,^13^,^22^,^23^.

Disturbance is another important driver of community assembly and beta diversity between assemblages^24^,^25^,^26^,^27^. The majority of forests in the world were disturbed by human activities^24^. Species in disturbed sites are under harsher environmental conditions than are undisturbed sites and thus are expected to favor pioneer species that can adapt to disturbed habitats. Theoretically, species in disturbed sites would phylogenetically be more strongly related than those in undisturbed sites. This prediction is supported by evidence found in several studies which showed that woody species in assemblages of earlier succession stages are more phylogenetically clustered^28^,^29^, suggesting that disturbed sites tend to select phylogenetically related species with similar ecological traits to tolerate disturbed site conditions. Following the same line of reasoning, it is expected that phylogenetic beta diversity would be lower between assemblages in disturbed sites, compared with those in undisturbed sites. Because abiotic filtering predominates during early succession while biotic filtering becomes increasingly important as succession proceeds^30^,^31^,^32^,^33^, it is expected that phylogenetic beta diversity is lower between assemblages at an early succession stage or in disturbed sites than those at a late succession stage or in undisturbed sites.

Subtropical broad-leaved forest is a major type of forests in the world^34^. In eastern Asia, the subtropical forest zone, which links tropical and temperate forest zones, cover an extensive area. For example, China alone possesses ~2.5 million km^2^ in the subtropical zone^34^. Subtropical broad-leaved forests in eastern Asia support a high biodiversity of the world, and their community structure and species composition are quite different from those in tropical and temperate forests^35^,^36^. Because the subtropical zone in China has sustained a high human population density for a long history, severe human disturbances have left few virgin forests in this region, and most forests were disturbed and currently are in the early- or mid-successional phases^24^,^34^. Previous studies have documented the effects of disturbance on forest species assembly and community phylogenetic structure^24^,^25^. However, to our knowledge, very few studies have simultaneously investigated taxonomic and phylogenetic turnover between tree species assemblages along environmental gradients in subtropical forests and how disturbance will influence taxonomic and phylogenetic beta diversity in these forests.

In this study, we intend to address the following three questions: 1) How do environmental and spatial variables contribute to the formation of taxonomic and phylogenetic beta diversity patterns of woody plants in subtropical forests? 2) Do beta diversity and underlying ecological processes differ between disturbed and undisturbed forests? 3) How are TBD, PBDt and PBDb metrics related to one another?

## Methods

### Study sites and data collection

**Figure 1:**
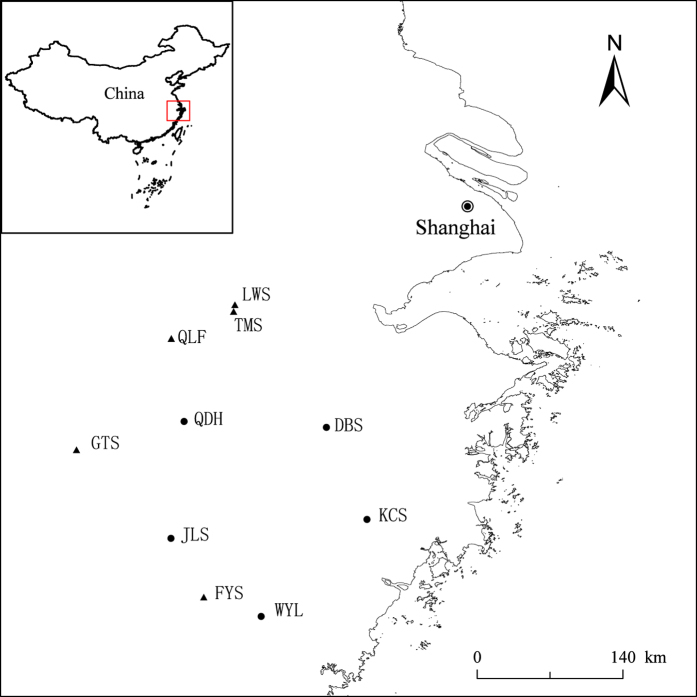
Location of the ten study forest plots in eastern China. Filled circles refer to disturbed forests, and filled triangles refer to undisturbed forests. The sites of DBS, FYS, GTS, JLS, KCS, LWS, QLF, WYL, TMS and QDH are located, respectively, in National Nature Reserve of Mount Dongbai, Mount Fengyang, Mount Gutian, Mount Jiulong, Mount Kuocang, Mount Longwang, Mount Qingliang, Wuyanling, Mount Tianmu, and Qiandaohu National Forest Park. Map was created with ArcGis, Version 10.0, http://www.arcgis.com/.

The study region is located in eastern China (Fig. 1). The region is under a subtropical monsoon climate. Mean annual temperature ranges from 15–18 °C among different years; mean annual precipitation ranges from 980–2000 mm, which mainly occurs from March to June (data from Weather China; www.weather.com.cn).

During the period from 2012 to 2013, ten 1-ha (100 m × 100 m) forest plots were established in the study region (Fig.1; Table S1). These plots were separated, on average, by 172.15 km, ranging from 6.61 to 300.52 km in distance. Data for disturbance history (i.e., years since last human disturbance) was collected from local authorities. We divided the 10 plots into two groups in terms of disturbance history. One group (treated as disturbed forests) included five secondary forests which were established on sites where forests were clear-cut 35 to 55 years ago (Table S1); the other group (treated as undisturbed forests) included five forests that have not been disturbed by humans for more than 150 years (Table S1). Plot establishment and data collection followed the protocol of the Center For Tropical Forest Science^37^. In each plot, all woody plants with diameter at breast height (DBH) ≥ 1 cm were tagged, identified and measured for DBH.

### Phylogenetic tree

**Figure 2:**
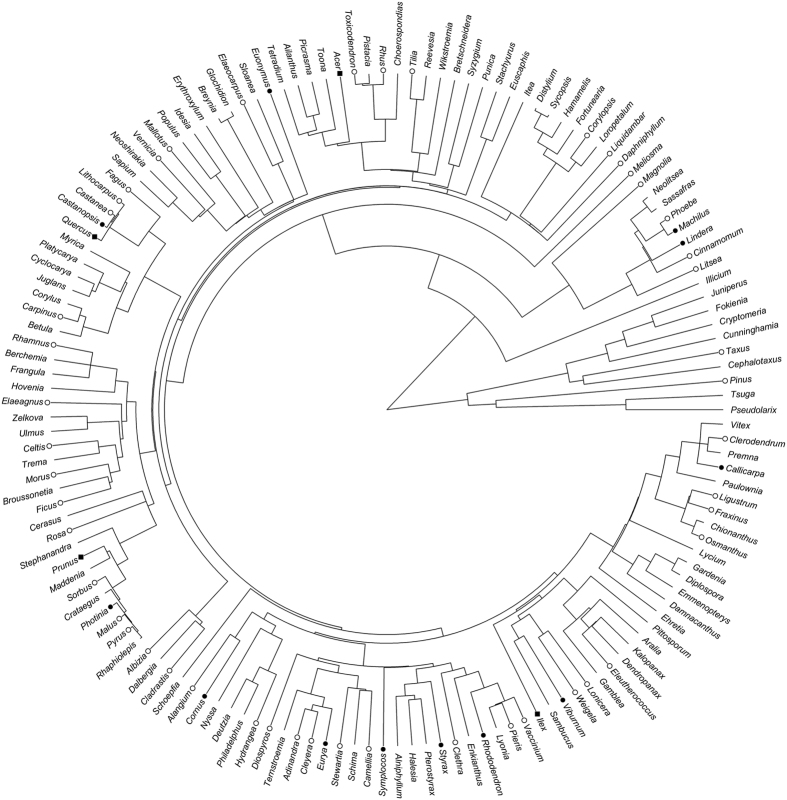
Phylogeny showing the 147 genera and their species richness in the studied plots (for illustrative purposes; analyses are based on a species-level phylogeny, see Materials and Methods). The number of species in each genus is indicated by symbols: tip with no symbol represents 1 species in the genus; open circle is 2–5 species; filled circle is 6–10 species; filled square is >10 species.

Botanical nomenclature of the species in the forest plots were standardized according to The Plant List (version 1.1; http://www.theplantlist.org/). The ten plots included 376 native tree species belonging to 61 families and 147 genera. We used genera and species present in our study plots to prune the mega-phylogeny PhytoPhylo^38^, which is an updated version of the phylogeny published in Zanne *et al*.^39^. This mega-phylogeny was constructed based on seven gene regions (i.e., 18S rDNA, 26S rDNA, ITS, *matK, rbcL, atpB,* and *trnL-F*), which include both slowly and quickly evolving regions^39^. The time scale for the phylogeny was based on 39 fossil calibrations^39^. Both minimum and maximum age constraints were utilized for each fossil calibration. All of the 61 families and 146 of the 147 genera in our study plots were included in PhytoPhylo. Thus, the phylogeny used in our study was completely resolved at the family level and nearly completely (99.3%) resolved at the genus level (Fig. 2). Of the 376 species present in our plots, 265 were also included in PhytoPhylo and additional 16 species belong to genera with only one species in our data and thus were represented by branches of their respective genera. Thus, the vast majority (75%) of the species present in our plots were resolved in the phylogeny extracted from PhytoPhylo. For the sole genus (i.e., *Cerasus*) and the remaining species of the study plots that were not in PhytoPhylo, we used the software S.PhyloMaker^38^ to add them to the phylogeny, using Scenario 3, which is analogous to using Phylomatic with BLADJ to generate a phylogeny^40^.

### Beta diversity indices

Previous studies suggest that multiple beta diversity indices be used in an analysis^15^,^16^ because different indices may emphasize on different aspects of community similarity and thus are complementary to each other to some degree. Accordingly, we used more than one beta diversity index for each of the two beta diversity categories (i.e., TBD vs. PBD) to quantify beta diversity among the ten forest plots. For TBD, we used Bray-Curtis and Jaccard indices^3^,^41^. PBD indices may be broadly divided into two classes: basal metric vs. terminal metric^15^. A basal metric of PDB emphasizes evolutionary divergence near the root node of the phylogenetic tree, which reflects historical events occurring in the remote past, whereas a terminal metric of PBD emphasizes evolutionary divergence near the terminal tips of the phylogenetic tree, which represent recent evolutionary events. Previous studies used PhyloSor, UniFrac and Dnn as terminal metrics and Dpw, Rao’s D and Rao’s H as basal metrics^15^,^16^,^42^. Because UniFrac and Rao’s D are redundant with PhyloSor and Dpw, respectively^16^, we used two terminal (PhyloSor, and Dnn) and two basal (Dpw and Rao’s H) metrics for PBD in the present study (Table S2). Larger values in Bray-Curtis, Jaccard, Dnn, Dpw and Rao’s H represent higher beta diversity.

### Geographical and environmental data

Geographical distance (*GeoDist*) between a pair of plots was measured as the Euclidean distance between the centers of the plots using DIVA-GIS software (http://www.diva–gis.org/). Environmental distance was measured from eight climatological parameters, which were extracted from the WORLDCLIM database (www.worldclim.org; for the period of 1950–2000 at a 30 arc-second resolution) using DIVA-GIS software^43^, including mean annual temperature (MAT), temperature seasonality (TS, i.e. standard deviation *100), maximum temperature of the warmest month (MTWM), minimum temperature of the coldest month (MTCM), annual precipitation (AP), precipitation of the wettest month (PWM), precipitation of the driest month (PDM) and precipitation seasonality (PS). Two additional climate-based parameters, annual actual evapotranspiration (AET) and annual potential evapotranspiration (PET), were obtained from the MODIS evapotranspiration data set (MOD16)^44^. Three topographic variables, elevation (ELE), aspect (ASP) and slope (SLOPE), were measured with a handheld global positioning system (GPS) receiver and clinometer. Aspect (*ASP*) is a circular variable and standardized using the formula of *–cos*((*2πASP*)/*360*) to make the maximum value at South and the minimum value at North so that it can be used in linear models (see Table S1).

### Statistical analysis

To disentangle the effect of environmental variables and spatial variables on beta diversity, two complementary approaches were used^45^. First, a principle component analysis (PCA) was performed on all environmental variables (i.e. standardized climatic and topographical variables) to capture differences in the environmental variables between plots (Table S1). Environmental distance (*EnvDist*) between plots was calculated as the Euclidean distance based on the first four principal components (PCs), which accounted for 91.07% of the total variance. Variance in disturbance history also calculated between plots (*AgeDist*). To estimate the relative importance of *EnvDist*, *GeoDist* and *AgeDist* on beta diversity metrics, we used the multiple linear regression model to assess the correlation between beta diversity and distance matrix^17^,^46^. We calculated the standardized regression coefficients using the “lm.beta” function in the R package QuantPsyc from the linear regression model.

Second, distance-based redundancy analysis (dbRDA) was conducted to assess the explanatory power of environmental variables, spatial variables on beta diversity of all forest plots as a whole as well as disturbed forests and undisturbed forests separately^47^. Seven spatial variables were obtained using the principal coordinates of neighboring matrices (PCNM) analysis^48^. Environmental variables included climate and topographic factors. Due to strong collinearity among some environmental factors, we removed environmental factors (e.g. ELE) that were primarily correlated with other factors (e.g., MAT) (Table S3). As a result, the selected environmental factors were MAT, AP, PET, ASP and SLOPE. We performed forward selection method (“forward.sel” function in the R package packfor 0.0–8) to select environmental, and PCNM variables that had significantly effects on beta diversity. The disturbance history was also considered into forward selection as environmental variable for all forest plots as whole. The significant explanatory environmental and PCNM variables retained in dbRDA were used to partition beta diversity into four fractions: variation explained by space only, variation explained by environment only, variation explained jointly by space and environment, and variation explained by neither.

We tested for differences in beta diversity between disturbed forests and undisturbed forests using a nonparametric analysis of Wilcoxon signed-ranks test. Mantel test was used to detect the correlation among beta diversity metrics. All analyses were conducted using R software (version 3.2.2, R Foundation for Statistical Computing, Vienna, Austria) with the packages of vegan, ecodist and picante.

Because gymnosperms have on average much longer evolutionary histories and thus longer branch lengths in a phylogeny than do angiosperms, including both gymnosperms and angiosperms in a phylogenetic analysis may obscure overall phylogenetic patterns^32^ even though gymnosperms accounted for only 3% of the species in our data set. Accordingly, we conducted two sets of analyses: one including both gymnosperms and angiosperms, the other including only angiosperms.

### Data availability

The data that support the findings of this study are available on request from the corresponding author (M.Y.). The data that we have used in this paper were collected through of joint projects of several research groups, and is the first time to be used for publishing in an international scientific journal. According to the agreement among the research groups who were involved in data collection, no research group can release the data until the projects finished. Thus, the agreement does not allow us to make the data to the public with our paper.

## Results

Regardless of whether an analysis included both gymnosperms and angiosperms or only angiosperms, multiple linear regression showed that AgeDist and GeoDist had no influence on beta diversity of all forests, EnvDist significantly correlated with TBD and PBDt metrics (*P* < 0.05 in both cases, except for the Jaccard metrics), but no variables were significantly correlated with PBDb metrics (Table 1; Table S4).

**Figure 3:**
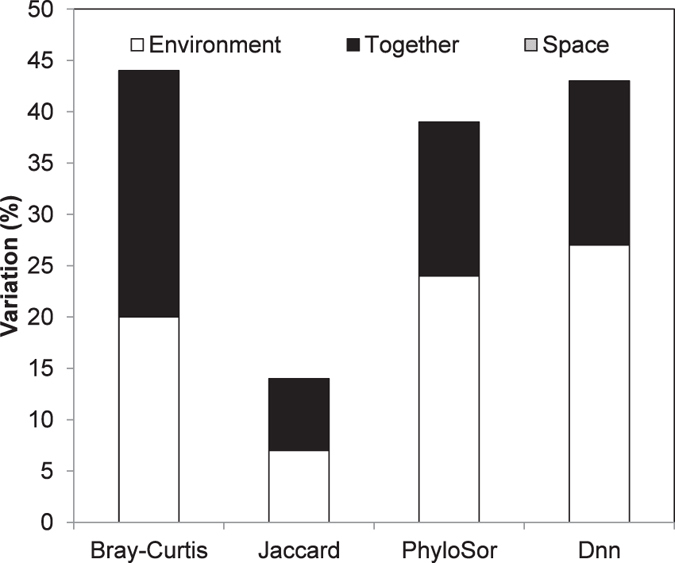
Variation in TBD (Bray-Curtis and Jaccard) and PBDt (PhyloSor and Dnn) explained by environmental and spatial variables with forward model selection for all forests. The beta diversity indices were calculated including both gymnosperms and angiosperms. The PBDb of Rao’s H and Dpw did not show in the plot, as no variables were selected by the forward selection for Rao’s H and variables explained little variance (less than 1%) for Dpw.

MAT and AP were the significant environmental variables (Table 2; Table S5), and together explained 7–27% of the variance in TBD and PBDt (Figs 3 and S1), whereas the pure spatial variable explained less than 1% of the variance in TBD and PBDt of all forests combined (Figs 3 and S1). The relative importance of environmental and spatial variables differed between disturbed and undisturbed forests (Table 2; Table S5). Bray-Curtis, PhyloSor and Dnn of disturbed forests were significantly affected by MAT (*P* < 0.05), but those of the undisturbed forests were determined by AP (Dnn: *R*_*adj*_ = 0.375, *P* < 0.05), topographical variable of SLOPE (Jaccard: *R*_*adj*_ = 0.094, *P* < 0.05) and spatial variable (e.g. Bray-Curtis: *R*_*adj*_ = 0.32, *P* < 0.05) (Table 2; Table S5). None of the environmental and spatial variables significantly explained variance in Rao’s H, and AP explained very little variance in Dpw (Table 2; Table S5). In general, the results from the analysis including both gymnosperms and angiosperms are consistent with those including only angiosperms (compare Table 2 with Table S5, and Fig. 3 with Figure S1).

**Figure 4:**
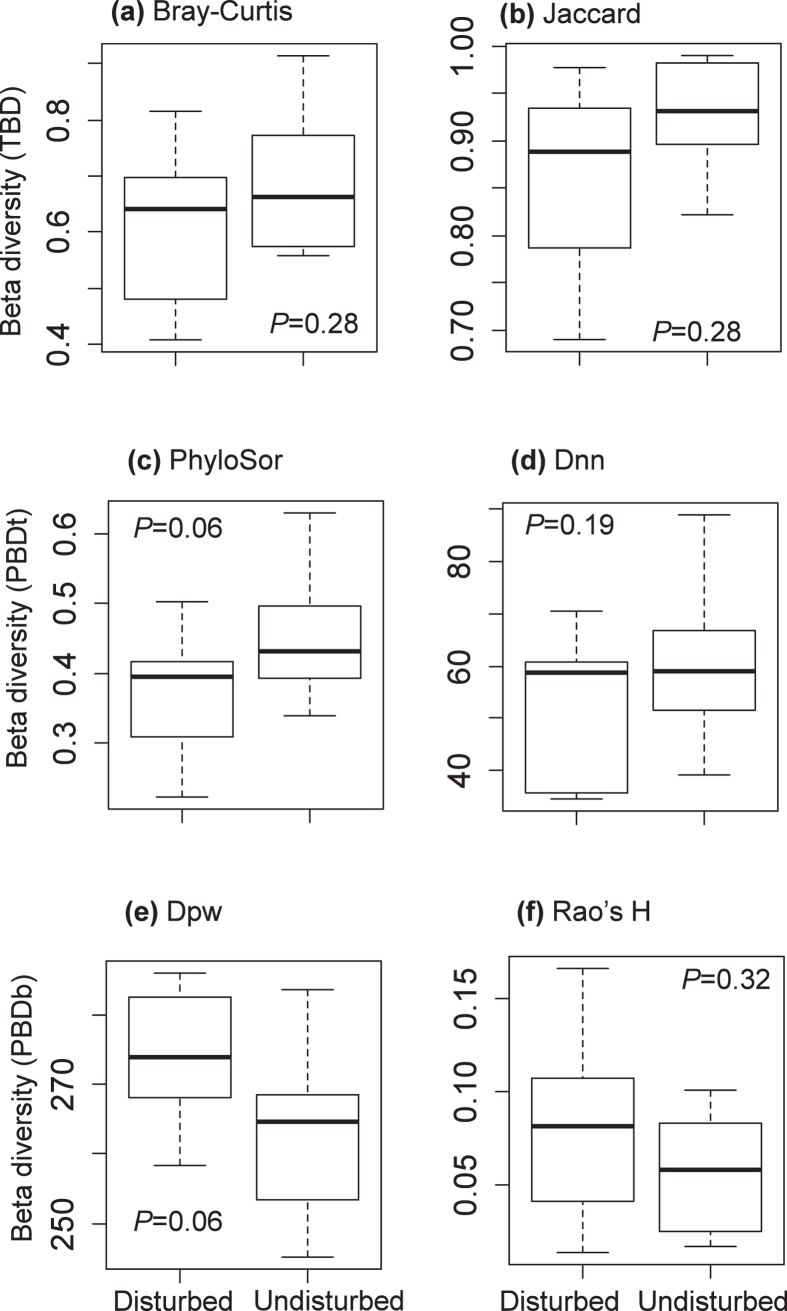
Comparison of beta diversity between disturbed forests and undisturbed forests. The beta diversity indices (i.e., Bray-Curtis, Jaccard, PhyloSor, Dnn, Dpw and Rao’s H) were calculated using both gymnosperms and angiosperms. Boxes represent the median and 25th/75th percentile, and whiskers extend to 1.5 times the interquartile range. *P* values were calculated by the nonparametric analysis of Wilcoxon singed ranks test.

Of the six indices of TBD and PBD, only PhyloSor and Dpw marginally significantly differed between disturbed and undisturbed forests (Wilcoxon test: *P* = 0.06; Fig. 4c,e) when both gymnosperms and angiosperms were considered (Fig. 4). Specifically, Dpw for disturbed forests was higher than that for undisturbed forests (Fig. 4e). In contrast, the PhyloSor was lower for disturbed forests (Fig. 4c). However, when only angiosperms were considered, there were no significant differences in TBD and PBD between disturbed and undisturbed forests except for Dpw (Wilcoxon test: *P* < 0.05; Fig. S2). Dpw was significantly higher for undisturbed forests than for disturbed forests (Fig. S2).

We found that the beta diversity metrics that were calculated using both gymnosperms and angiosperms were significantly correlated with the beta diversity metrics that were calculated using only angiosperms (mantel test: *r* ≥ 0.615, *P* < 0.01) except for Dpw (*r* = −0.164, *P* > 0.1). TBD metrics were significantly correlated with PBDt metrics (*r* ≥ 0.757, *P* < 0.001) (Table 3; Table S6). The Rao’s H metric was correlated with the metrics of Jaccard (*r* = 0.557, *P* < 0.01), Dnn (*r* = 0.552, *P* < 0.05) and Dpw (*r* = 0.610, *P* < 0.05); and the Dpw metric was only correlated with Rao’s H metric when both gymnosperms and angiosperms were considered (Table 3).

## Discussion

The drivers of taxonomic and phylogenetic beta diversity include both dispersal limitation and environmental filtering. Understanding which drivers are more important in regulating patterns of beta diversity can provide insights into the mechanisms underlying community assembly^3^,^14^,^21^,^22^. Most studies on comparison of taxonomic and phylogenetic beta diversity and their relationships with environmental (i.e., niche process) and spatial (i.e., neutral process) variables for local forest communities were conducted in temperate and tropical forests. To our knowledge, the present study is the first to simultaneously compare taxonomic and phylogenetic beta diversity for forest communities at a regional extent in a subtropical region, to compare beta diversity between disturbed and undisturbed forests using a variety of beta diversity indices quantifying both shallow and deep histories of evolution, and to relate beta diversity with environmental and spatial distances. We have found some patterns, which we discuss below.

### Contributions of space and environment to beta diversity

The main aim of our study was to assess which processes structure the beta diversity in subtropical forests. The result of multiple regression analysis shows that environmental distance was significantly correlated with TBD and PBDt metrics (Table 1), whereas the geographical distance and disturbance history have no influence on TBD and PBDs (Table 1), suggesting that environmental filtering plays a key role in structuring taxonomic and closely related species assembly in the subtropical forests. Generally, closely related species are likely to share more similar trait values than that expected under a Brownian motion model of trait evolution^16^,^49^. Our finding that environmental distance between forests have played a more important role in driving beta diversity than geographic distance is contrary to that of Saito, *et al*.^50^, who found that geographical distance, rather than environmental distance, better explained patterns of phylogenetic beta diversity in Neotropical stream meta-communities. However, our finding is consistent with the finding of Hardy *et al*.^11^ who found that spatial distance does not have a significant effect on phylogenetic turnover of trees in tropical forests when accounting for rainfall. These studies suggest that dispersal limitation does not contribute to taxonomic and phylogenetic turnover in some study systems at a regional scale^11^. The importance of environment in driving beta diversity among the subtropical forests studied is also consistent with previous studies which found that environmental filtering has played a strong role on selecting plant traits in subtropical forests^51^,^52^,^53^.

Furthermore, if distance generating geographic isolation is a strong force in causing the regional pattern of species distribution, patterns at shallow levels in the phylogeny (i.e., PBDt) might be influenced by spatial distance; alternatively, if geographic isolation causing dispersal limitation is a strong force in forming the regional species pool, patterns of PBD emphasizing deep nodes in the phylogeny (i.e., PBDb) might be influenced by spatial distance^14^. The result found in this study that spatial distance had no power in explaining PBD (Table 1; Fig. 3) suggests that dispersal limitation might not be an important factor in determining the spatial distribution of the study species in the present study. It is also possible that the relatively small sample size (i.e., 10 sites) of our study has a limited power in detecting the effect of spatial distance on beta diversity, although similar sample sizes have been used in previous studies (e.g., 12 sites were used in study of Culmsee and Leuschner^54^).

The present study showed that disturbance history has no power in explaining variation in TBD and PBD, suggesting that local scale processes, such as succession and dispersal, might not be important in determining the taxonomic and phylogenetic turnover in community composition among regional forests, although they might be important in determining the taxonomic and phylogenetic community structures within these forests^24^.

Previous studies found that environmental variables related to temperature and/or precipitation drive community assembly of temperate forests^12^,^22^,^55^. While dispersal limitation and/or environmental filtering can both be the key determinants of beta diversity in tropical forests^10^,^42^, in subtropical forests, previous studies conducted at local scales found that space and environment were both related to beta diversity and their relative importance depended on spatial scale or sampling size^53^,^56^. According to these findings, environmental filtering seems to become more important from tropical to temperate areas, which might be due to geographic variation in temperature and precipitation^3^,^57^. Similar to the findings in temperate forests^22^, our study indicated that MAT and AP were the environmental variables that explained the most variation of TBD and PBDt (Table 2), which supports the notion that temperature and precipitation limit the distribution of tree species in eastern China^58^.

Moreover, there is still a large amount of the variation in TBD and PBDs unexplained in this study. We suspect this may be due to a combination of local stochastic processes (e.g., ecological drift) underlying community assembly and/or unmeasured environmental and spatial variables^48^,^56^. Especially, soil characteristic may be important in determining species composition in subtropical forests^53^ and should be considered in future studies.

### Beta diversity in disturbed and undisturbed forests

The second major goal of the present study was to compare beta diversity in disturbed and undisturbed forests and to determine the relative importance of environmental and spatial variables on beta diversity. Our results indicate that taxonomic beta diversity between disturbed and undisturbed forests did not significantly differ regardless of which beta diversity index was used and whether gymnosperms were considered. However, when phylogenetic beta diversity was quantified using PhyloSor and Dpw, phylogenetic beta diversity differed substantially between disturbed forests and undisturbed forests when both gymnosperms and angiosperms were considered. Feng *et al*.^24^ found that past tree harvesting could affect subtropical forest structure by promoting the establishment of certain light-demanding and pioneer species. Our results support the notion that environmental filtering is the main process in driving the beta diversity patterns of the studied forests. We might find that the species composition will be more similar among disturbed forests, as the habitat environment become more homogeneous after clear-cut. Tree establishment will generally shift from shade-intolerant, short-lived pioneer trees and shrubs in disturbed forests to shade-tolerant, long-lived pioneers and wind-pollinated species in undisturbed forests^59^. The relative importance of environmental and spatial variables will change in disturbed and undisturbed forests when habitat heterogeneity in these forests change. As we showed in this study, TBD and PBDt of disturbed forests were only explained by MAT, while in undisturbed forests they were determined by AP, slope and spatial variable (Table 2). This may explain why PhyloSor for gymnosperms and angiosperms combined and Dpw for only angiosperms in disturbed forests were lower than those in undisturbed forests (Figs 4 and S2). However, Dpw for disturbed forests was higher than that for undisturbed forests when both gymnosperms and angiosperms were considered (Fig. 4e). This may be because gymnosperms such as *Pinus massoniana* are pioneer species and are often dominant trees in disturbed forests. Because gymnosperms have on average much longer branch lengths in a phylogeny than do angiosperms, phylogenetic turnover at more basal nodes will be higher than that at more terminal nodes.

### Correlations among beta diversity metrics

The beta diversity metrics that reflect different evolutionary depths have different sensitivities to detect the beta diversity along environmental and spatial gradients. In this study, we found that PBDt metrics (PhyloSor and Dnn) were significantly correlated with TBD metrics (Bray-Curtis and Jaccard) (Table 3). This finding is consistent with those of previous studies^15^,^16^,^42^. The high correlation between PBDt and TBD may suggest that more recent radiations exist in the study region, and PBDt and TBD will be similarly tippy if taxonomic species’ relationships are represented as a polytomous star-like phylogenetic tree^16^. However, because PBDb is sensitive to turnover deeper in the phylogeny^15^,^42^ and many dominant tree species belong to the same genera (e.g., *Ilex*, *Quercus* and *Prunus*) (Fig. 2)^36^, the PBDb may have less power to detect the effect of environmental and spatial variables in this region. In addition, the Dpw that was calculated using both gymnosperms and angiosperms was not correlated with Dpw for which only angiosperms were considered; as a result, whether gymnosperms are included in an analysis would substantially change the result of the analysis, as shown in the present study.

Overall, we investigated the drivers of taxonomic and phylogenetic beta diversity in subtropical forests in eastern China. We found that factors of environmental filtering, mainly temperature and precipitation, played a significant role in determining TBD and PBDt of the studied forest communities. In addition, we found that the climate variable of temperature played an important role in disturbed forests, whereas the climate, topographical and spatial variables all played an important role in driving beta diversity in undisturbed forests. TBD and PBDt were slightly higher in undisturbed forests than in disturbed forests. Our results would deepen our understanding of the processes that underlie the taxonomic and phylogenetic beta diversity in subtropical forests, which is vital to understanding the relative importance of niche and neutral process in driving community assembly of forests from tropical toward temperate regions.

## Additional Information

**How to cite this article**: Liu, J. *et al*. Disentangling the drivers of taxonomic and phylogenetic beta diversities in disturbed and undisturbed subtropical forests. *Sci. Rep.* **6**, 35926; doi: 10.1038/srep35926 (2016).

## Supplementary Material

Supplementary Information

## Figures and Tables

**Table 1:** Results of multiple linear regression models.

	GeoDist	EnvDist	AgeDist	*R*^*2*^_adj_	*F*	*P*
Bray-Curtis	0.181	0.346^*^	0.045	0.161	3.817	<0.05
Jaccard	−0.006	0.271	0.091	0.029	1.438	0.245
PhyloSor	0.104	0.427^**^	0.108	0.219	5.111	<0.01
Dnn	0.034	0.343^*^	0.124	0.111	2.822	<0.05
Rao’s H	0.078	0.277	0.106	0.068	2.076	0.118
Dpw	0.118	−0.396^*^	0.133	0.056	1.879	0.148

The significance for standardized regression coefficients and adjusted *R-*square (*R*^*2*^_*adj*_) were calculated in models with environmental distance (EnvDist), geographical distance (GeoDist) and the variance of forests disturbance history (AgeDist) as explanatory variables and beta diversity matrices as the response variable. Both gymnosperms and angiosperms were included in the beta diversity matrices. Significance level: ^**^P ≤ 0.01, ^*^0.01 < *P* ≤ 0.05.

**Table 2:** Environmental and spatial variables selected by the forward selective procedure in the RDA (*P* ≤ 0.05) in all, disturbed and undisturbed forests.

		Variables	All	Disturbed	Undisturbed
Bray-Curtis	Environment	MAT	0.188^**^	0.408^*^	
		AP	0.394^**^		
	Space	PCNM1	0.167^**^		0.318^*^
Jaccard	Environment	AMT	0.061^**^		
		AP	0.118^*^		
		SLOPE			0.094^*^
	Space	PCNM1	0.042^*^		
PhyloSor	Environment	MAT	0.189^***^	0.434^*^	
		AP	0.353^**^		
	Space	PCNM1	0.129^*^		
Dnn	Environment	MAT	0.269^**^	0.491^*^	
		AP	0.389^*^		0.375^*^
	Space	none	—		
Rao’s H	Environment	none	—		
	Space	none	—		
Dpw	Environment	AP	0.010^*^		0.043^*^
	Space	PCNM1			0.017^*^

Values refer to the cumulative adjusted *R*^*2*^ (adjR^2^Cum) of the variables selected. The beta diversity indices were calculated using both gymnosperms and angiosperms in each site. MAT: mean annual temperature; AP: annual precipitation; SLOPE: slope and PCNM1: PCNM variable. Significance level: ^***^P ≤ 0.001, ^**^0.001 < *P* ≤ 0.01, ^*^0.01 < *P* ≤ 0.05.

**Table 3:** Pearson’s correlation coefficients based on mantel test (999 permutations) among community dissimilarity metrics.

	Bray-Curtis	Jaccard	PhyloSor	Dnn	Rao’s H	Dpw
Jaccard	0.858^***^	1.000				
PhyloSor	0.924^***^	0.779^***^	1.000			
Dnn	0.927^***^	0.805^***^	0.935^***^	1.000		
Rao’s H	0.408^#^	0.557^*^	0.306	0.552^*^	1.000	
Dpw	0.100	0.245	–0.022	0.210	0.610^*^	1.00

The beta diversity indices were calculated using both gymnosperms and angiosperms in each site. Significance level: ^***^P ≤ 0.001, ^**^0.001 < *P* ≤ 0.01, ^*^0.01 < *P* ≤ 0.05, ^#^0.05 < *P* ≤ 0.1.
