# Supplementary material for: Disentangling the drivers of taxonomic and phylogenetic beta diversities in disturbed and undisturbed subtropical forests
Source: Sci Rep. 2016 Oct 24;6:35926. doi: 10.1038/srep35926 (PMC5075936; doi:10.1038/srep35926)
Supplement: Supplementary Information [file srep35926-s1.pdf]

Disentangling the drivers of taxonomic and phylogenetic beta diversities in disturbed and undisturbed subtropical forests

Jinliang Liu, Hong Qian, Yi Jin, Chuping Wu, Jianhua Chen, Shuquan Yu, Xinliang Wei, Xiaofeng Jin, Jiajia Liu & Mingjian Yu

Supplementary Information

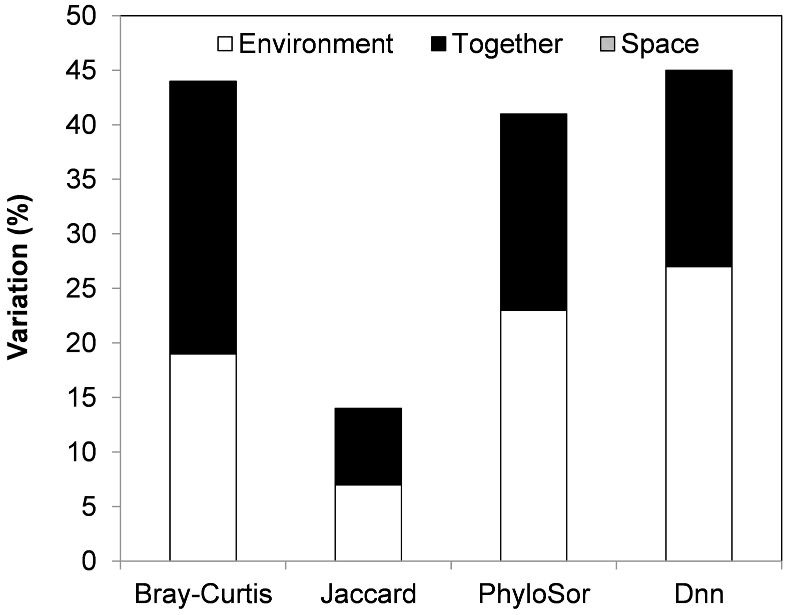

Figure S1 Variation in TBD (Bray-Curtis and Jaccard) and PBDt (PhyloSor and Dnn) explained by environmental and spatial variables with forward model selection for all forests. The beta diversity indices were calculated using angiosperms in sites. The BPDb of Rao's H and Dpw did not show in the plot, as no variables were selected by the forward selection for Rao's H and variables explained little variance (less than 1%) for Dpw.

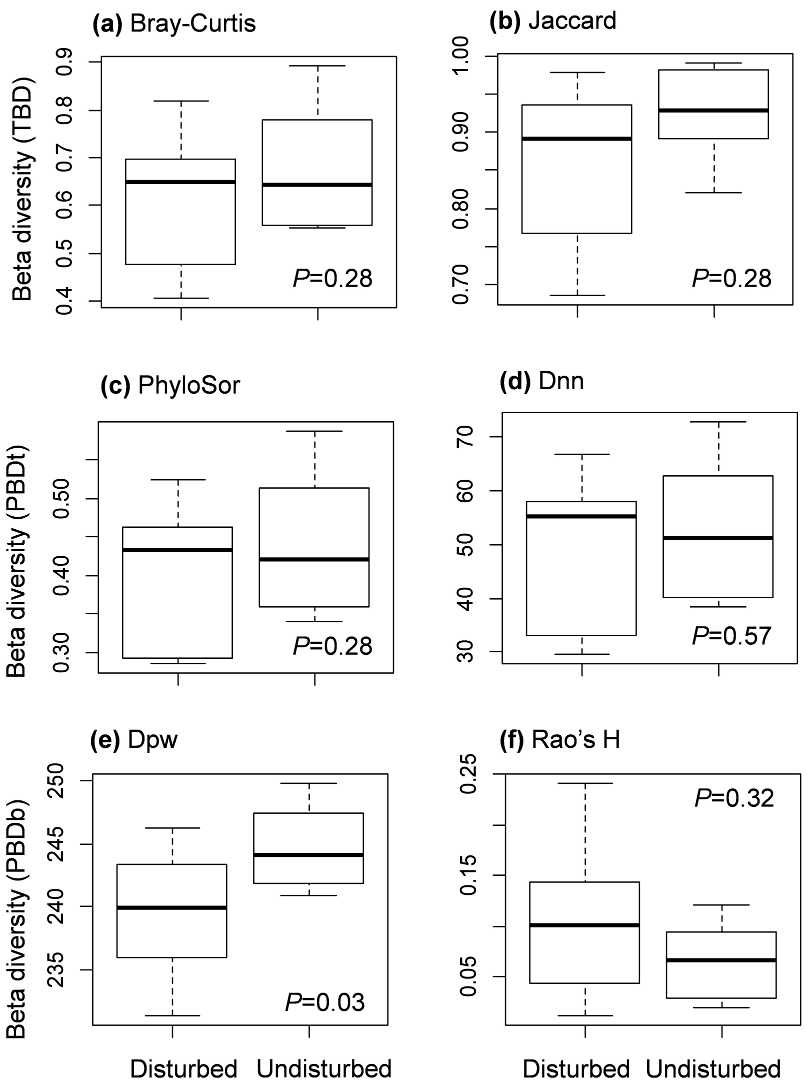

Figure S2 Comparison of beta diversity between disturbed forests and undisturbed forests. The beta diversity indices (i.e., Bray-Curtis, Jaccard, PhyloSor, Dnn, Dpw and Rao's H) were calculated using angiosperms. Boxes represent the median and 25th/75th percentile, and whiskers extend to 1.5 times the interquartile range. *P* values were calculated by the nonparametric analysis of Wilcoxon singed ranks test.

Table S1 Information of the ten study plots. MAT: mean annual temperature (℃); TS: temperature seasonality (STD*100); MTWM: max temperature of warmest month (℃); MTCM: min temperature of coldest month (℃); AP: annual precipitation (mm); PWM: precipitation of wettest month (mm); PDM: precipitation of driest month (mm); PS: precipitation seasonality; AET: annual actual evapotranspiration (mm); PET: annual potential evapotranspiration (mm); ASP: aspect; ELE: elevation (m) and AGE: forest disturbance history, 1 refers to the forests were clear-cut about 35 ~ 55 years ago and 2 refers to the forests undisturbed by human at least 150 years.

	No. of species	No. of individuals	MAT	TS	MTWM	MTCM	AP	PWM	PDM	PS	AET	PET	ASP	SLOPE	ELE	AGE
DBS	46	5395	16.33	865.99	32.7	0.4	1424	221	43	47.11	761.1	1408.5	0.75	33.5	146	1
FYS	109	7087	11.78	696.24	24.6	-2.0	2119	337	47	51.78	736.1	1438.0	0.54	35.0	1544	2
GTS	101	5289	16.30	848.58	31.7	0.3	1818	311	52	58.39	962.6	1464.2	0.99	35.0	683	2
JLS	127	5333	14.34	770.44	28.5	-0.6	1953	331	46	53.09	820.7	1463.4	-0.87	37.0	747	1
KCS	100	3085	15.30	791.05	30.0	0.5	1742	253	45	50.38	861.1	1401.9	-0.98	27.3	428	1
LWS	99	4776	10.81	830.80	25.7	-4.6	1618	244	46	49.82	767.5	1442.0	-0.99	21.8	1315	2
QLF	102	5876	12.21	837.95	27.1	-3.2	1732	280	48	50.28	764.0	1383.3	0.98	38.5	975	2
QDH	52	10867	16.70	886.05	33.0	0.6	1537	264	47	53.04	798.7	1402.7	-0.78	36.5	150	1
TMS	118	3042	12.10	846.07	27.3	-3.5	1566	242	46	49.75	762.1	1364.6	0.79	30.0	1069	2
WYL	130	7473	14.56	720.23	28.2	0.4	1895	299	42	51.41	758.8	1412.4	0.64	30.0	960	1

Table S2 Beta diversity indices used in this study.

Index	Formula	Description	Reference
Bray–Curtis	$1-\frac{2\times S_{k_1k_2}}{S_{k_1}+S_{k_2}}$	Calculates species dissimilarity among communities.	Koleff *et al.* 2003
Jaccard	$1-\frac{S_{k_1k_2}}{S_{k_1}+S_{k_2}-S_{k_1k_2}}$	Calculates species dissimilarity among communities.	Koleff *et al.* 2003
PhyloSor	$1-\frac{2\times BL_{k_1k_2}}{BL_{k_1}+BL_{k_2}}$	Calculates phylogenetic dissimilarity among communities.	Swenson 2011
Dnn	$\frac{\sum_{i=1}^{S_{k_1}}\min\delta_{ik_2}+\sum_{j=1}^{S_{k_2}}\min\delta_{jk_1}}{2}$	Calculates the mean nearest phylogenetic neighbor between all species in two communities.	Swenson 2011
Dpw	$\frac{\sum_{i=1}^{S_{k_1}}\overline{\delta_{ik_2}}+\sum_{j=1}^{S_{k_2}}\overline{\delta_{jk_1}}}{2}$	Calculates the mean pairwise phylogenetic distance between all species in one community to all species in another community.	Swenson 2011
Rao's H	$D_{kl}-(D_{kk}+D_{ll})/2$	Standardized measure of phylogenetic distinctness.	Webb *et al.* 2008

S_{k1k2} is the number of species shared between community k_1 and k_2, S_{k1} and S_{k2} are the numbers of species in community k_1 and k_2, respectively. BL_{k1k2} is the total length of the branches shared between community k_1 and k_2, BL_{k1} and BL_{k2} are the total branch lengths in in community k_1 and k_2, respectively. min δ_{jk1} is the phylogenetic distance between species *j* in community k_2 and its nearest species in community k_1. $\overline{\delta_{ik_2}}$ is the mean pairwise phylogenetic distance between species *i* in community k_1 to all species in community k_2 and $\overline{\delta_{jk_1}}$ is the mean pairwise phylogenetic distance between species *j* in community k_2 to all species in community k_1. If an ultrametric phylogeny is supplied, D_{kk} is equivalent to the mean pairwise phylogenetic distance between two individuals drawn from community *k*, D_{kl} is the mean pairwise phylogenetic distance between individuals drawn from community *k* and community *l*.

Table S3 Pearson's correlation coefficients among environmental variables. Significance is indicated in the upper triangle of the table. MAT = mean annual temperature; TS= temperature seasonality (STD*100); MTWM= max temperature of warmest month; MTCM= min temperature of coldest month; AP= annual precipitation; PWM= precipitation of wettest month; PDM= precipitation of driest month; PS= precipitation seasonality; AET= annual actual evapotranspiration; PET= annual potential evapotranspiration; ASP=aspect; SLOPE=slope; ELE=elevation. Significance level: ***$P \leq 0.001$, **$0.001 < P \leq 0.01$, *$0.01 < P \leq 0.05$, #$0.05 < P \leq 0.1$ and — not significant.

	MAT	TS	MTWM	MTCM	AP	PWM	PDM	PS	AET	PET	ASP	SLOPE	ELE
MAT	1.00	—	***	***	—	—	—	—	—	—	—	—	***
TS	0.329	1.00	#	—	***	*	—	—	—	—	—	—	#
MTWM	0.946	0.611	1.00	*	—	—	—	—	—	—	—	—	***
MTCM	0.932	-0.030	0.77	1.00	—	—	—	—	#	—	—	—	*
AP	-0.255	-0.868	-0.534	0.040	1.00	***	—	—	—	#	—	—	#
PWM	-0.071	-0.668	-0.323	0.145	0.927	1.00	—	*	—	*	—	—	—
PDM	0.001	0.296	0.047	-0.142	0.182	0.364	1.00	*	—	—	—	—	—
PS	0.328	-0.036	0.209	0.318	0.449	0.653	0.751	1.00	**	*	—	—	—
AET	0.548	0.257	0.504	0.454	0.077	0.202	0.632	0.767	1.00	#	—	—	—
PET	0.119	-0.297	-0.019	0.190	0.518	0.605	0.342	0.594	0.459	1.00	—	—	—
ASP	-0.036	-0.017	-0.038	-0.046	0.054	0.100	0.167	0.054	-0.068	-0.252	1.00	—	—
SLOPE	0.347	0.059	0.283	0.332	0.230	0.485	0.360	0.345	0.097	0.043	0.382	1.00	—
ELE	-0.892	-0.586	-0.953	-0.736	0.577	0.415	0.125	0.003	-0.364	0.146	0.184	-0.255	1.00

Table S4 Results of multiple linear regression models. The significance for standardized regression coefficients and adjusted *R*-square were calculated in models with environmental distance (EnvDist), geographical distance (GeoDist) and the variance of forests disturbance history (AgeDist) as explanatory variables and beta diversity matrices as the response variable. Only angiosperms were included in the beta diversity matrices. Significance level: $^{*}0.01<P\leq0.05$.

	GeoDist	EnvDist	AgeDist	R^2_{adj}	*F*	*P*
Bray-Curtis	0.199	0.321*	0.027	0.145	3.485	<0.05
Jaccard	0.010	0.239	0.075	0.008	1.124	0.351
PhyloSor	0.169	0.328*	0.094	0.155	3.699	<0.05
Dnn	0.078	0.277	0.106	0.068	2.076	0.118
Rao's H	–0.083	–0.055	0.042	0.000	0.182	0.908
Dpw	0.206	–0.044	0.054	0.000	0.584	0.629

Table S5 Environmental and spatial variables selected by the forward selective procedure in the RDA (*P* ≤ 0.05) in all, disturbed and undisturbed forests. Values refer to the cumulative adjusted R^2 (adjR^2Cum) of the variables selected. The beta diversity indices were calculated using angiosperms. Significance level: $^{***}P \leq 0.001$, $^{**}0.001 < P \leq 0.01$, $^{*}0.01 < P \leq 0.05$

		Variables	All	Disturbed	Undisturbed
Bray-Curtis	Environment	MAT	0.179***	0.400*	
		AP	0.388**		0.254*
	Space	PCNM1	0.173**		0.33*
Jaccard	Environment	MAT	0.059*		
		AP	0.115*		
		SLOPE			0.092*
	Space	PCNM1	0.046*		
PhyloSor	Environment	MAT	0.182***	0.427*	
		AP	0.377**		0.316*
	Space	PCNM1	0.147**		
Dnn	Environment	MAT	0.263***	0.487*	
		AP	0.402*		0.419***
	Space	none	--		
Rao’s H	Environment	none	--		
	Space	none	--		
Dpw	Environment	AP			
		SLOPE		0.013**	
	Space	PCNM1			

Table S6 Pearson's correlation coefficients based on mantel test (999 permutations) among community dissimilarity metrics. The beta diversity indices were calculated using only angiosperms in each site. Significance: $^{***}P\leq0.001$, $^{**}0.001<P\leq0.01$, $^{*}0.01<P\leq0.05$, $^{\#}0.05<P\leq0.1$

	Bray-Curtis	Jaccard	PhyloSor	Dnn	Rao’s H
Bray-Curtis	1.000				
Jaccard	0.848***	1.000			
PhyloSor	0.939***	0.805***	1.000		
Dnn	0.931***	0.803***	0.954***	1.000	
Rao’s H	0.386$^{\#}$	0.530**	0.422*	0.577**	1.000
Dpw	0.123	–0.005	–0.018	–0.003	–0.380

Reference

Koleff, P., Gaston, K. J. & Lennon, J. J. Measuring beta diversity for presence–absence data. *J. Anim. Ecol.* **72**, 367-382 (2003).

Swenson, N. G. Phylogenetic beta diversity metrics, trait evolution and inferring the functional beta diversity of communities. *PLoS One* **6**, e21264 (2011).

Webb, C. O., Ackerly, D. D. & Kembel, S. W. Phylocom: software for the analysis of phylogenetic community structure and trait evolution. Bioinformatics **24**, 2098-2100 (2008).
